# Navigating the landscape of mitochondrial-ER communication in health and disease

**DOI:** 10.3389/fmolb.2024.1356500

**Published:** 2024-01-23

**Authors:** Conor T. Ronayne, Pedro Latorre-Muro

**Affiliations:** ^1^ Department of Cancer Biology, Dana-Farber Cancer Institute, Boston, MA, United States; ^2^ Department of Cell Biology, Harvard Medical School, Boston, MA, United States

**Keywords:** mitochondria, endoplasmic reticulum, mitochondrial-ER communication, cellular fitness, protein homeostasis, unfolded protein response, lipid trafficking, calcium signaling

## Abstract

Intracellular organelle communication enables the maintenance of tissue homeostasis and health through synchronized adaptive processes triggered by environmental cues. Mitochondrial-Endoplasmic Reticulum (ER) communication sustains cellular fitness by adjusting protein synthesis and degradation, and metabolite and protein trafficking through organelle membranes. Mitochondrial-ER communication is bidirectional and requires that the ER-components of the Integrated Stress Response signal to mitochondria upon activation and, likewise, mitochondria signal to the ER under conditions of metabolite and protein overload to maintain proper functionality and ensure cellular survival. Declines in the mitochondrial-ER communication occur upon ageing and correlate with the onset of a myriad of heterogeneous age-related diseases such as obesity, type 2 diabetes, cancer, or neurodegenerative pathologies. Thus, the exploration of the molecular mechanisms of mitochondrial-ER signaling and regulation will provide insights into the most fundamental cellular adaptive processes with important therapeutical opportunities. In this review, we will discuss the pathways and mechanisms of mitochondrial-ER communication at the mitochondrial-ER interface and their implications in health and disease.

## Introduction

Organisms are influenced by several environmental factors such as environmental temperatures (Latorre-Muro et al., 2021), physical activity, diseases, or diet (Sohn et al., 2023). Continuous and dynamic metabolic adjustments allow organismal adaptations to these environmental cues. The landscape of intracellular interactions is diverse across the different cellular organelles. In eukaryotes, the interplay between the endoplasmic reticulum (ER) and mitochondria (mitochondrial-ER communication) is a central node for the control of cellular bioenergetics. Mitochondrial-ER communication can also be more tightly established by the physical interaction between these two organelles by series of protein-protein contacts that create a scaffold for signal transduction and metabolite trafficking. These physical contacts were described in the late 1950s (Copeland and Dalton, 1959) and form the Mitochondrial-ER contacts (MERCs) that connect mitochondria to the ER at the mitochondrial-associated membranes (MAMs). Importantly, mitochondrial-ER communication is disrupted in several age-related diseases such as cancer (Wang et al., 2015), obesity and type 2 diabetes (Koh et al., 2007; Yuzefovych et al., 2013; Arruda et al., 2014), or neurological disorders (Hedskog et al., 2013; Sepulveda-Falla et al., 2014; Lau et al., 2020). Consequently, the maintenance of mitochondrial-ER communication ensures the execution of appropriate cellular responses upon external stimuli and the maintenance of organismal health.

Mitochondrial-ER communication in response to environmental factors is dictated by the activation of key regulators of the Integrated Stress Response residing in the ER. These effectors include the Inositol-requiring transmembrane kinase/endoribonuclease 1α (IRE1α), Activating Transcription Factor 6 (ATF6) and Protein kinase R (PKR)-like endoplasmic reticulum kinase (PERK) (Harding et al., 1999). IRE1α, ATF6 and PERK have been shown to regulate mitochondria and cellular biogenetics through transcriptional pathways such as IRE1α-XBP1, ATF6 cleavage, or the PERK-eIF2α-ATF4 axis (Harding et al., 1999; Harding et al., 2000), respectively, which promote the activity of the electron transport chain (ETC) in mitochondria and oxidative metabolism (Balsa et al., 2019; Kato et al., 2020; Latorre-Muro et al., 2021). More recently, non-canonical roles of these signal transducers also suggest a direct intervention in triggering protein-activation cascades or physically interact with ER or mitochondrial partners at the MAMs. For instance, IRE1α and ATF6 can regulate calcium signaling to mitochondria through the inositol 3-phosphate receptor (IP_3_R) (Bartok et al., 2019; Carreras-Sureda et al., 2019; Carreras-Sureda et al., 2022), which is essential to activate oxidative metabolism. Additionally, IRE1α activity is modulated by outer-mitochondrial membrane (OMM) E3 ubiquitin ligase MARCH5 linking mitochondrial ER contacts to unfolded protein responses (Takeda et al., 2019). Further, IRE1α is linked to the mitochondrial apoptotic machinery, where caspase 3 and 7 cleave IRE1α leaving a luminal domain fragment that inhibits BAX oligomerization in the ER and subsequent translocation to the OMM (Shemorry et al., 2019). Consistent with this, the Unfolded Protein Response (UPR) modulates cellular responses to proteotoxic stress in the mitochondrial inner membrane space, and heighted levels of ER stress result in dissociation of mitochondria from ER-mitochondria encounter structures (ERMES) in yeast (Kakimoto-Takeda et al., 2022). Further, PERK has been shown to regulate cristae dynamics aside from its canonical pathway by controlling the mitochondrial import machineries (Latorre-Muro et al., 2021) and lipid trafficking (Sassano et al., 2023). Therefore, MERCs provide dual signaling routes that synchronize nuclear-transduced signals with rapid and local adaptive responses that regulate mitochondrial function.

The effectiveness of cellular metabolic adaptations relies on the plasticity of mitochondria to fine-tune its activities. Mitochondria were so-called as the “powerhouses” of the cell and, more recently, several works have indicated their role in a myriad of processes beyond the supply of energy including stress sensing, antigen recognition and immunity (Lin et al., 2010; Gordon et al., 2020), nucleotide homeostasis (Bennett et al., 2021), and ER stress (Takeda et al., 2019), as representative examples. The vast majority of mitochondrial proteins (99%) are encoded by nuclear genes that need to be translated at the cytosolic ribosomes and translocated through the mitochondrial membranes to their final location. Import of mitochondrial precursors into mitochondria depends on the Translocase of the Outer Mitochondrial membrane 40 (TOM) complex at the OMM which can quickly incorporate newly synthesized precursors through co-translational import mechanisms (Williams et al., 2014). For outer mitochondrial proteins, insertion is also granted by cytosolic chaperones, the SAM50 machinery (Diederichs et al., 2020), and MTCH1/2 insertases (Guna et al., 2022). Intermembrane and inner mitochondrial membrane proteins follow a path to their final location through the Translocase of the Inner Mitochondrial membrane (TIM) complexes. These processes are crucial to ensure mitochondrial function and require delicate regulation to avoid the clogging and collapse of protein trafficking across membranes. Quality-control processes sustain mitochondrial-ER communication through different mechanisms including the surveillance and storage of competent precursors prior to their import, unclogging (Weidberg and Amon, 2018), and degradation of non-viable precursors (Peterson et al., 2019; Wu et al., 2020; Pisa and Rapoport, 2022). In addition, mitochondrial translation of mitochondrial-encoded genes that are key components of the OXPHOS machineries need to pair their activity to that at the cytosolic ribosomes. Retrograde communication from mitochondria to the ER also adjust the strength of the ISR responses to support cellular adaptation and survival (Perry et al., 2021; Ronayne et al., 2023). For instance, inhibition of mitochondrial translation can increase cell survival *in vivo* and *in vitro* by attenuating the stress response from the IRE1α pathway (Soustek et al., 2018; Ronayne et al., 2023). The landscape of protein-protein contacts that occur at the MERCs is at the intersection of several signaling pathways that connect mitochondrial-ER communication and transduce signals to other organelles.

In this review, we will discuss the mechanisms of mitochondrial-ER communication that allow cellular adaptation and survival. We will focus on the subset of protein interactions and signaling pathways upon external stimuli that act as messengers between the ER and mitochondria and shape the nature of the adaptive responses at the ER-OMM crossroad.

## Protein translation at the MERCs crosstalks with mitochondrial protein synthesis

**FIGURE 1:**
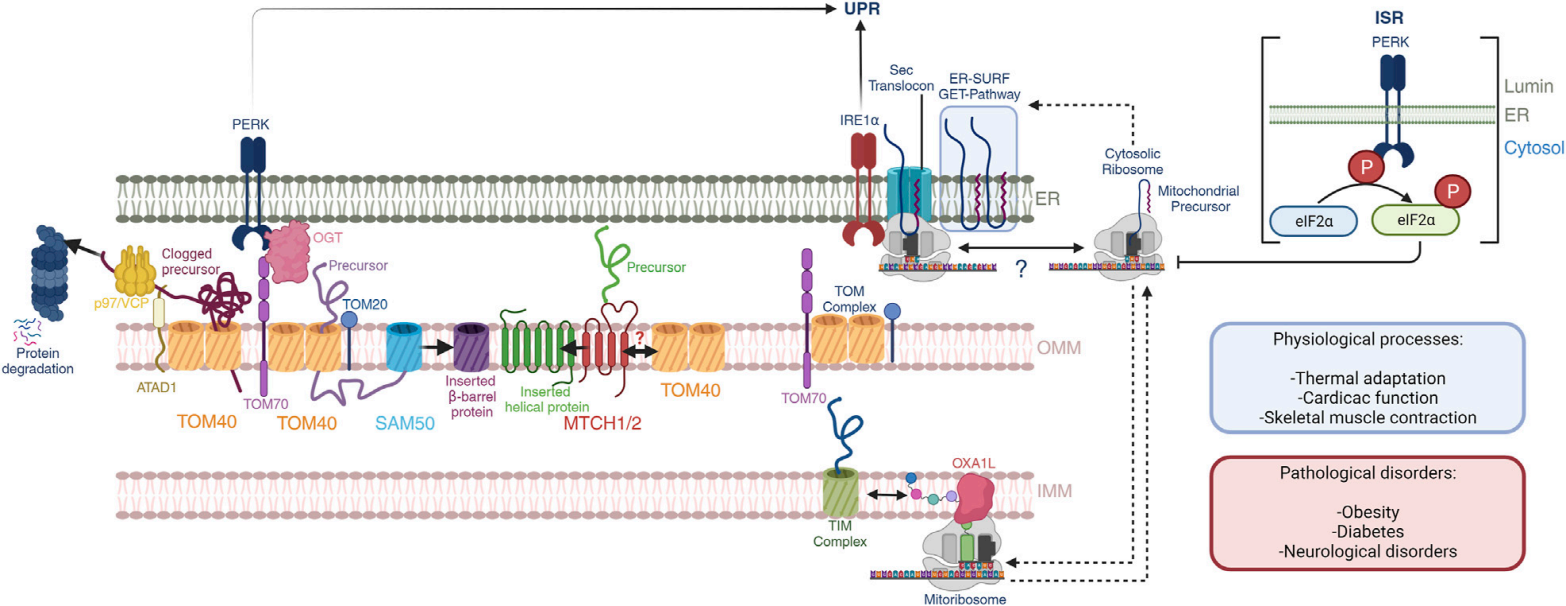
Mitochondrial-ER communication regulates protein homeostasis. OMM protein biogenesis is regulated by the insertases MTCH1/2 for α-helical proteins and the tandem TOM40-SAM50 for β-barrel proteins. Nuclear encoded mitochondrial proteins routed towards the intermembrane space, IMM and mitochondrial matrix are imported through the TOM40 pore at the OMM, which is facilitated by OMM receptors TOM70 and TOM20 through the binding of mitochondrial targeting sequences. PERK activation regulates TOM70 activity and dependent mitochondrial protein import through OGT. Quality control mechanisms in mammals at the OMM is dependent on the activity of the OMM AAA-containing ATPase ATAD1, which extracts clogged proteins and presents them to p97/VCP with subsequent proteosome degradation. Import and assembly of nuclear-encoded mitochondrial proteins is coordinated with mitochondrial-encoded proteins. The mitoribosome co-translationally inserts nascent peptides into the IMM through a direct interaction with the OXA1L insertase. Nuclear-encoded proteins that have been imported into the intermembrane space are further translocated into the IMM through the TIM complexes. Here, nascent peptides derived from both nuclear and mitochondrial genomes are assembled into ETC complexes. Further, mitochondrial and cytosolic translation rates are coordinated during mitochondrial biogenesis, and the ER membrane serves as a buffer of mitochondrial targeted peptides through the GET- and ER-SURF pathways during this process. In this context, the translation rates and ER-association capacity of cytosolic ribosomes may be dictated by mitochondrial translation. These processes may impact the UPR through the proximity of IRE1α to the Sec translocon. Additionally, the PERK ISR attenuates cytosolic translation through direct phosphorylation of eIF2α, affecting mitochondrial precursors, and potentially their presence at the ER-membrane. These pathways play important roles in normal physiological processes including thermal adaptation, cardiac function, and skeletal muscle contraction, to name a few. Defects herein are associated with obesity, diabetes, and neurological disorders.

Mitochondrial-ER communication requires an exquisite control over the protein synthesis pathways at the ribosomal level. Initial electron microscopy studies identified the ultrastructure of the ER in the 1950s (Porter, 1953) and classified into rough and smooth. The apparent roughness of the ER was later found to be the association of the cytosolic ribosomes where nuclear encoded genes are translated and proteins folded, processes that require quality controls. Likewise, mitochondrial ribosomes translate mitochondrial encoded genes. Co-regulation of the translation of mitochondrial nuclear-encoded precursors and translation of mtDNA derived OXPHOS genes is crucial for mitochondrial functions such as proteostasis and respiration. Here we will present the mechanisms of cross-communication between the cytosolic and mitochondrial protein translation machineries in adaptive and disease processes (Figure 1).

Regulation of cellular proteostasis at the level of cytosolic translation is most widely appreciated through the activation of the mTORC1 pathway which largely governs catabolic and anabolic states of the cell (Bennett et al., 2022a; Bennett et al., 2022b). Sensory inputs include growth factors and amino acids which mTORC1 senses to adjust cytosolic translation, metabolism, and autophagy. Direct targets of mTORC1 include eukaryotic translation initiation factor 4E (eIF4E)-binding protein 1 (4E-BP1) and ribosomal protein S6 kinase beta-1 (S6K1) which together promote translation upon mTORC1 activation (Bennett et al., 2022a; Bennett et al., 2022b). Specifically, S6K1 phosphorylates the eukaryotic translation initiation factor 4B (eIF4B) to activate the 5′ cap-binding eIF4F complex. In parallel, 4E-BP1 phosphorylation disrupts 4E-BP1-eIF4E complex which leads to enhanced 5′-cap-dependent translation of mRNAs. In a mechanistically parallel fashion, PERK controls translation by phosphorylating eIF2α, which results in global translation attenuation of 5′ cap-dependent translation, and specific translation of mRNAs with upstream open reading frames that code for genes involved in cell survival including ATF4, ATF5, CHOP, and GADD34 (Bennett et al., 2022a; Bennett et al., 2022b). Intriguingly, the PERK-ATF4 axis regulates mitochondrial super-complex assembly under nutrient stress conditions linking the ISR to mitochondrial respiration and cristae formation (Balsa et al., 2019). Further, IRE1α is directly associated to the Sec-translocon (Li X. et al., 2020) and its activation can be modulated by the extent of cytosolic ribosome co-translational translocation into the ER lumen which tunes the ER protein loading capacity in coordination with cytosolic translation. Translational control, whether it be mTOR or ISR regulation has been implicated in pro-survival mechanisms in a variety of diseases including cancer where targeting mTOR and PERK have been proposed as new generation therapeutic strategies in different contexts. The role of the mitochondria and concomitant MERCs in the regulation of global translation processes is an area of current interest in the field and representative examples of mitochondrial and cytosolic translation regulation will be presented in the coming sections.

To expand on these regulatory mechanisms in light of mito-nuclear translation systems, recent studies have highlighted potential co-regulatory mechanisms of cytosolic and mitochondrial ribosomes (mitoribosomes). mtDNA encodes 13 hydrophobic subunits of the ETC complexes that are necessary for respiratory function and provide essential structure and function for the assembly and activity of nuclear encoded complex subunits (Kummer and Ban, 2021). Mitochondrial polycistronic mRNAs are translated by mitoribosomes in cooperation with a variety of initiation, elongation, and termination factors in a GTP-dependent fashion (Brown et al., 2017; Khawaja et al., 2020; Kummer and Ban, 2021; Itoh et al., 2022; Remes et al., 2023). This process is carried out with the large mitoribosome subunit tethered to the mitochondrial inner membrane by a direct interaction with the insertase OXA1L (Itoh et al., 2021). Peptide elongation is coupled with co-translational insertion of hydrophobic subunits that are efficiently inserted into the inner membrane (Itoh et al., 2021). In parallel, nuclear-encoded mitochondrial subunits are imported, processed, and assembled with mitochondrial subunits in a tightly controlled receptor- and chaperone mediated process; to be further detailed in a subsequent section of this review. In this regard, cytosolic and mitochondrial translation systems are coordinated to ensure proper stoichiometry under dynamic and changing nutrient environments; tuning respiratory processes in cooperation with ISR and UPR (ex. PERK, IRE1α, ATF6) components accordingly.

### Balance of mitochondrial and nuclear-derived proteins in mitochondrial biogenesis

Specific regulators of mito-nuclear balance are still being uncovered, but recent studies identified potential candidate factors (Isaac et al., 2018). Cytosolic and mitochondrial ribosome profiling in yeast has identified that translation programs are highly coordinated across these sub-organelle compartments during mitochondrial biogenesis (Couvillion et al., 2016). During yeast adaptation to mitochondrial respiratory growth conditions, cytosolic and mitochondrial ribosomes preferentially translate mRNAs encoding complex III and IV over complex V subunits, demonstrating a specific coordination of translation systems in the context of mitochondrial proteins (Couvillion et al., 2016). Interestingly, this regulation is seemingly unidirectional and is communicated from cytosol to the mitoribosome, but not reverse, as inhibition of mitoribosomes did not alter cytosolic translation rates of mitochondrial precursors (Couvillion et al., 2016). Specifically, LRPPRC, an RNA binding protein required for mitochondrial mRNA stability in mammals, sustains mitochondrial translation (Ruzzenente et al., 2012; Soto et al., 2022), where CRISPR depletion of LRPPRC results in decreased mitochondrial translation without changes in cytosolic protein synthesis (Soto et al., 2022). Here, imbalanced cytosolic translation results in the accumulation of mitochondrial precursors when compared to mitochondrial derived proteins causing a proteostasis defect (Soto et al., 2022). As mentioned above, cytosolic and mitochondrial translation systems require an intricate balance of stoichiometry to avoid protein mis-localization and improper complex biogenesis, where the ISR and UPR sense and respond to resulting protein stresses. The nuclear-mitochondrial imbalance following LRPPRC deletion triggers unfolded protein responses by activating ER stress sensors IRE1α and ATF6, and UPR/ISR transcription factor ATF4 (Soto et al., 2022), further illustrating the benefit of localized protein translation and mitochondrial import in close proximity to the ER. The importance of LRPPRC in maintaining mitochondrial translation and coordinating proteostasis between the mitochondria, cytosol, and ER highlights a complex organization across compartments that may require cross-talk between mitochondrial translation factors, cytosolic protein synthesis, and ER stress responses.

To expand on this concept, a mammalian cellular model with unbalanced mito-nuclear complex IV subunits was used to perform a genome-wide CRISPR activation screen which identified two factors, PREPL and NME6 that control the biogenesis of complex IV (Kramer et al., 2023). Here, PREPL promotes complex IV biogenesis by modulating mitochondrial lipid homeostasis and mitochondrial protein synthesis, where NME6 is involved in sensing mitochondrial RNA abundance, mitoribosome assembly and RNA pseudouridylation (Kramer et al., 2023). In fact, PREPL deficiency has been associated with a recessive metabolic disorder that result in neonatal hypotonia, growth hormone deficiency, and feeding difficulties (Regal et al., 2018; Rosier et al., 2021). Specifically, PREPL KO mice exhibit reduced mitochondrial complex function and abnormal ultrastructure’s (Rosier et al., 2021). These studies provide further insights into potential factors involved in the maintenance of mito-nuclear balance and potentially their underlying translation systems that when dysregulated can result in pathology.

TRAP1, a mitochondrial matrix 90 kDa chaperone, has also been identified as a factor coordinating mitochondrial and cytosolic protein elongation (Avolio et al., 2023). Initially described as a chaperone stabilizing PPIF (Peptidyl-prolyl isomerase F) and inhibiting apoptosis through the permeability transition pore (Kang et al., 2007), the role of TRAP1 related physiology is now largely appreciated in mitochondrial respiration. Specifically, TRAP1 is a member of the mitochondrial HSP90 chaperone family, and contributes to the maintenance of respiration in cells, where it has been implicated in binding mitochondrial and cytosolic ribosomes along with core mitochondrial import TOM40 complex (Avolio et al., 2023). Here, TRAP1 binds and activates TOM40-mediated import in a mitochondrial translation-dependent manner which is also influenced by cytosolic translation (Avolio et al., 2023). Intriguingly, TRAP1 localizes to the MAM compartment (Amoroso et al., 2012; Avolio et al., 2023) where its binding to cytosolic ribosomes, import machinery, and mitochondrial translation system are in close proximity to the ER where protein translation, import, and degradation can be dynamically monitored by the UPR. The involvement of TRAP1 in numerous aspects of mito-nuclear balance offers an additional example of chaperone-mediated translational control that supports protein homeostasis at MAMs. It can be reasoned that the dynamic expression of TRAP1 and related chaperones can respond to challenging microenvironments supporting protein homeostasis in the context of mitochondrial biogenesis in normal physiology and disease.

### Mitoribosome quality control and ER stress responses

Quality control mechanisms that mediate mitochondrial translation fidelity in response to mitoribosome elongational stalling have recently been described (Desai et al., 2020; Kummer and Ban, 2021). In one context, elongational stalling in response to aberrant tRNA accommodation results in the recruitment of rescue (mtRF-R and MTRES1) and assembly/stability factors (MALSU1, LOR8F8 and mtACP1) (Desai et al., 2020). Recently, our laboratory discovered that mitoribosome targeting agents, including tetracyclines, can rescue mitochondrial mutant cells from nutrient deprivation (Perry et al., 2021). Mechanistically, we found that tetracyclines activate mitoribosome quality control and recruit MALSU1 to the large mitoribosome subunit (Ronayne et al., 2023). This MALSU1 recruitment was found necessary to promote survival at the tetracycline-inhibited mitoribosome and illustrated that mitoribosome quality control can dictate cell fate under stress conditions (Ronayne et al., 2023). Mitochondrial mutant cells can also be rescued with pharmacological inhibition of the UPR through IRE1α (Soustek et al., 2018). We found that tetracyclines attenuate UPR signaling in complex I mutant cells, and this attenuation was sufficient to rescue these cells from cell death (Ronayne et al., 2023). Interestingly, attenuation of the UPR with tetracyclines required mitoribosome quality control factor MALSU1, consistent with its necessity for cell survival (Ronayne et al., 2023). Interestingly, these survival responses were independent of known mitochondrial ISR signaling through DELE1 or ATF4, or mitochondrial outer membrane E3-ligase MARCH5 which attenuates IRE1α oligomerization and activation (Takeda et al., 2019; Fessler et al., 2020; Guo et al., 2020; Ronayne et al., 2023). Thus, the identification of MALSU1’s role in communicating stress responses to the ER illustrates a novel role of mitochondrial translation in dictating proteostasis and cell death responses in the context of bioenergetic and protein stress. These results further support the findings on mitoribosome translation factor LRPPRC where its KO results in proteostasis defects in the cytosol and activation of the UPR (Soto et al., 2022).

## Mitochondrial protein import at the MERCs

The success of mitochondrial-ER adaptive responses relies on lipid and protein remodeling across cellular organelles (Bennett et al., 2021). This is particularly important in mitochondria as the main metabolic regulator in the cell that need to adjust their activities to satisfy cellular energetic demands. To sustain protein homeostasis and mitochondrial function during environmental stress, synthesized proteins (Williams et al., 2014) are stored (Kramer et al., 2023), transported (Wiedemann and Pfanner, 2017), and/or also degraded (Weidberg and Amon, 2018). Proteins are incorporated into different mitochondrial compartments including the outer mitochondrial membrane (OMM), the intermembrane space (IMS), the inner mitochondrial membrane (IMM) or the matrix (Wiedemann and Pfanner, 2017). In response to environmental cues, mitochondrial functions are modulated, and their proteome remodeled which raises the fundamental question on how protein trafficking is regulated in mitochondria. Indeed, physiological evidence indicates that the import machineries are a control checkpoint that responds to the signals transduced by the ER upon cellular stress (An et al., 2019; Latorre-Muro et al., 2021). The pathways driving the mitochondrial precursors to their proper location are distinct and have been exhaustively reviewed before (Wiedemann and Pfanner, 2017). Here, we will discuss their roles in context of physiology and disease.

### Protein insertion at the OMM

The incorporation of proteins to the outer mitochondrial membrane is possible through three main pathways: i) spontaneous insertion (Doan et al., 2020), ii) chaperone-driven (Drwesh et al., 2022) and/or iii) insertase-driven (Becker et al., 2011; Diederichs et al., 2020; Guna et al., 2022). The biochemical nature of the different proteins defines the pathways utilized. Two distinct protein conformations populate the OMM, β-barrel proteins such as TOM40 or SAM50 and α-helical components including TOM70 or TOM20.

β-barrel proteins require specialized machineries to facilitate their insertion. Mim1 controls the insertion of signal anchored and β-barrel proteins which further favor the insertion of OMM proteins (Becker et al., 2011; Drwesh et al., 2022). However, no mammalian ortholog of the Mim complex is known. Yet, β-barrel protein insertion at the OMM is possible through the Sorting Assembly Machinery (SAM50 complex) (Kozjak et al., 2003), a conserved complex in mammals, yeast and bacteria (BAM complex (Han et al., 2016)). SAM50 is a β-barrel protein that forms a 7–9 nm pore that allows the transport of precursors (Diederichs et al., 2020) and participates in the insertion of Voltage-Dependent Anion-selective Channel (VDAC) or TOM40 (Hohr et al., 2018). Interestingly, TOM40 cooperate with SAM50 facilitating the translocation of β-barrel precursors to the IMS prior to their processing by SAM50 and final insertion at the OMM (Qiu et al., 2013). SAM50 also contacts with the Mitochondrial Contact Site and Cristae Organizing System (MICOS) complex at the IMM providing a structural support to maintain mitochondrial architectural integrity (Tang et al., 2020). Proteomic survey studies in human skeletal muscle show decreased levels of SAM50 in obese and diabetic individuals compared to lean subjects (Hwang et al., 2010). Consistently, deletion of SAM50 negatively impacts mitochondrial function brown adipocyte thermogenic functions (Park et al., 2022) which are tightly dependent on ER signaling (Kato et al., 2020; Latorre-Muro et al., 2021). These findings connect β-barrel OMM protein insertion with cellular fitness and the onset of metabolic diseases (Guo et al., 2013; Xue et al., 2019; Tang et al., 2020).

The insertion of α-helical subunits can be spontaneous due to the thermodynamically favorable accommodation of the hydrophobic transmembrane domains upon their embedding to the OMM (Doan et al., 2020). Yet, this process may be inefficient and at the risk of protein aggregation. In yeast, cytosolic chaperones Ydj1 and Sis1 can assist the insertion of signal-anchored OMM proteins (Drwesh et al., 2022) however, these chaperone-assisted mechanisms need further exploration in a pathophysiological context in mammals. Importantly, mammalian MTCH2 and its paralog MTCH1 can act as insertases for helical OMM proteins (Guna et al., 2022). MTCH1/2 were charactered as members of the SLC25 mitochondrial carrier family, which typically reside at the IMM except for MTCH1/2 which are located at the OMM (Ruprecht and Kunji, 2020). MTCH1/2 are an import scaffold for multiple types of α-helical OMM proteins (tail-anchored, signal-anchored and multipass membrane proteins) including essential import receptors such as TOM70, TOM20 (discussed below). Absence of the MTCH1/2 insertase increases the accumulation of mitochondrial precursors in the ER membranes which likely safeguards from the deleterious consequences of misfolded precursor proteins at the MAMs and signals to the ER to adjust protein synthesis and prevent the overload of the import machineries (Guna et al., 2022). MTCH1 is also known as Presenilin-1 Associated Protein (PSAP) and has a proapoptotic role and is involved in Alzheimer’s disease (Lamarca et al., 2007), connecting mitochondrial insertases with the development of neurodegenerative disorders. Additionally, MTCH2 positively controls oxidative function in mitochondria (Buzaglo-Azriel et al., 2016) and its locus has been associated with the development of metabolic diseases such as obesity and diabetes (Fall et al., 2012; Fischer et al., 2023). While the involvement of mitochondrial protein import by mammalian MTCH1/2 and the development of pathologies needs further study, it is clear that the understanding of the mechanisms of OMM insertion may provide new therapeutical opportunities for metabolic and age-related diseases.

### Protein translocation through the TOM pore

The incorporation into mitochondria for intermembrane space (IMS) and inner mitochondrial membrane (IMM) proteins is possible through the TOM40 complex (Wiedemann and Pfanner, 2017). TOM40 is a β-barrel protein associated with TOM22, TOM5, TOM6 and TOM7 (TOM complex) which has been typically described forming dimers, and more recently trimers and tetramers (Tucker and Park, 2019). Higher order oligomers of the TOM complex correlate with increased import and their formation is facilitated by TOM6 (Sakaue et al., 2019) but decreased by TOM7 (Becker et al., 2011) indicating that the entry of precursor proteins to mitochondria can be regulated by varying TOM complex subunit stoichiometries. This balance might need fine tuning since TOM7 loss-of-function can, however, cause severe growth and developmental defects (Garg et al., 2022). Each TOM40 β-barrel subunit displays a 40Å wide pore that allows the entry of unfolded mitochondrial precursors (Tucker and Park, 2019). Defective import crosstalks with the ER components. For instance, blockage of the TOM complex in mouse adipose tissue upon expression of a mitochondrial-targeted amyloid protein results in an obesity and diabetes (An et al., 2019) likely due to severe mitochondrial dysfunction that may lead to the activation of ER-stress responses (Koh et al., 2007). Similarly, mutants of the mitochondrial adenine nucleotide transporter (ANT) (Simoncini et al., 2017) arrest mitochondrial protein import and associate with the development of muscular and neurological disorders (Coyne et al., 2023). On the other hand, the ER can control protein trafficking through the import machineries. In mammals, PERK exemplifies this regulation by controlling the activity of the Translocase of the Inner Mitochondrial Membrane (TIM) complexes (Rainbolt et al., 2013) and the TOM70 pathway (Latorre-Muro et al., 2021) (see discussion below) connecting mitochondrial-ER communication and protein trafficking through the OMM and IMM. These studies highlight the relevance of mitochondrial protein import in the maintenance of mitochondrial homeostasis which otherwise degenerates in pathologies.

The TOM complex is tightly dependent on the activity of two accessory receptors TOM20 (Dekker et al., 1998; Su et al., 2022) and TOM70 (Young et al., 2003; Zanphorlin et al., 2016) with cytosolic domains that are exposed to the ER membrane. Both receptors recognize TOM complex substrates and provide a means to stabilize them in the unfolded state prior to their import. TOM20 mainly recognizes proteins with a cleavable N-terminal mitochondrial targeting sequence (MTS) while TOM70 shows affinity for proteins with internal hydrophobic domains such as the mitochondrial solute carriers and proteins prone to aggregation (Backes et al., 2021). TOM20 and TOM70 have overlapping functions and the lack of one of them can be compensated to some extend by the other subunit (Fan et al., 2011). In addition, cooperative binding between TOM20 and TOM70 indicates that both pathways are not exclusive and can participate in the import of the same precursors at different stages (Fan et al., 2011). In mammals, both receptors are central to the mitochondrial-ER adaptive responses triggered by environmental factors. There is little information on how the TOM20 pathway is controlled in response to environmental stressors at the MERCs. In contraction-stimulated muscle fibers, TOM20 protein abundance is increased and correlates with higher levels of mitochondrial malate dehydrogenase (MDH2) (Takahashi et al., 1998) yet is unclear whether this is due to mitochondrial biogenesis through transcriptional coactivators such as PGC1α (Puigserver et al., 1998) among others. TOM20 can act as an oxidative stress sensor in melanoma cells although its impact on mitochondrial protein import is unclear (Zhou et al., 2018).

In mammals, the OMM receptor TOM70 is a target of the PERK-branch of the ISR to promote cristae formation and increase mitochondrial-dependent nutrient oxidation (Latorre-Muro et al., 2021). Studies in brown adipocytes show that PERK positively controls mitochondrial biogenesis and function during cold acclimation (Balsa et al., 2019; Kato et al., 2020; Latorre-Muro et al., 2021). Brown adipocytes are specialized cells that in response to cold environments, increase the mitochondrial oxidation of glucose (Orava et al., 2011), fatty acids (Lee et al., 2016) or branched-chain amino acids (Yoneshiro et al., 2019) to generate heat and maintain body temperature in mammals (Cannon and Nedergaard, 2004). Brown adipose tissues are activated through the stimulation of β3-adrenergic receptors with the neurotransmitter norepinephrine and initiate series of transcriptional, translational and post-translational programs that ultimately lead to mitochondrial remodeling (Kato et al., 2020; Latorre-Muro et al., 2021), increased nutrient oxidation and heat production (Cannon and Nedergaard, 2004). Mitochondrial remodeling is prevalent after a week in a cold environment and can be observed within the first 24–48 h after cold stimulation (Kato et al., 2020; Latorre-Muro et al., 2021). Physical activity (Richard and Rivest, 1989; Bostrom et al., 2012) or high-calorie diets (Feldmann et al., 2009) can also activate brown adipose tissue development or function, respectively, with the purposes of maintaining organismal glucose homeostasis and body weight. Therapeutically, brown adipocytes are a target to treat obesity and associated diseases (Yoshida et al., 1994; Valentine et al., 2022). Failure to activate PERK results in defective thermogenesis and thus, the ability to maintain body temperature constant (Kato et al., 2020; Latorre-Muro et al., 2021). Mechanistically, PERK initiates a post-translational modification cascade that augments the activity of the TOM70 receptor and the import of the cristae component MIC19 to promote cristae formation (Latorre-Muro et al., 2021). Similarly, increased TOM70 protein expression in fasted livers promotes cristae formation and protects against diet-induced obesity through the MIC19 cristae component (Sohn et al., 2023). Interestingly, diet-induced obesity increases mitochondrial-ER contacts in hepatocytes likely due to aberrant ER reorganization in an attempt to compensate the lack of communication with mitochondria (Arruda et al., 2014). Chemical induction of mitochondrial-ER contacts has a detrimental effect in hepatocytes suggesting that a balance in the number of contacts regulates cellular homeostasis in an hormetic fashion (Arruda et al., 2014).

Therefore, the transduction of stress signals from the ER to mitochondria can dictate the dynamics of mitochondrial protein import and provide new mechanisms to understand the regulation of these machineries in a pathophysiological context.

## Protein quality control at the MERCs

Under conditions of cellular stress, the overload of unfolded proteins needs specific processing to avoid the accumulation of undesirable protein conformations that can negatively impact cellular functions. In fact, 30% of recently translated proteins need to be removed due to improper folding. This phenomenon can also happen at the MAMs and hamper the adaptive capability of the cells. The ER is one of the main stores of non-imported mitochondrial precursors to avoid the saturation of the import machineries. The ER-SURF, ERMES, and the ERAD systems are quality control mechanism that surveil the MAMs space to facilitate the transduction of stress signals from the ER to mitochondria. Here, we will focus on the protein quality control mechanisms at the MAMs and their involvement in pathophysiological processes.

The ERMES complex is a molecular tether that joins the OMM and ER in yeast (Kornmann et al., 2009). This complex was initially identified from a genetic screen and further detailed using genome-wide mapping strategies to identify 4 proteins Mmm1/Mdm10/Mdm12/and Mdm34 that are resident in both ER and mitochondrial compartments (Kornmann et al., 2009). Functionally, the ERMES complex is involved in the transfer of Ca^2+^ and lipids (Kornmann et al., 2009), processes that will be further discussed in the subsequent sections. Recently, the cryo-EM structure of the ERMES complex has been solved providing details into the mechanism of ER-mitochondria lipid transfer through synaptotagmin-based lipid-protein binding domains at these contact sites (Wozny et al., 2023). To expand on the role of protein-protein interactions and MERCs, a recently discovered pathway details the role of the ER in buffering mitochondrial proteins on the ER-membrane surface (ER-SURF) (Hansen et al., 2018). Using a genome-wide screen in yeast, the ER membrane and more-specifically, the membrane chaperone Djp1, mediates the delivery of mitochondrial targeted proteins that accumulate in the ER during cytosolic translation (Hansen et al., 2018). Although the precise targeting mechanisms of mitochondrial precursors to the ER-membrane are being discovered, the guided-entry of tail-anchored (GET) protein pathway has been implicated in inserting mitochondrial tail-anchored proteins in the ER membrane (Xiao et al., 2021). Additionally, a recent chaperone-mediated mechanism has been described in yeast where accumulating mitochondrial precursors in the cytosol are sequestered by heat-shock proteins Hsp42 and Hsp104 in transient complexes termed MitoStores (Kramer L. et al., 2023). These complexes protect the translocation pore from clogging, and from the toxic accumulation of precursors in the cytosol. Here, it can be reasoned that the activity of the proteosome, and also the capacity of the ER and UPR signaling can play a cooperative role in dictating both ER-SURF, GET, and MitoStore based pathways, and the close proximity of this signaling at MERCs is important for its fidelity. In fact, decreased UPR and proteosome activity, along with mitochondrial integrity are factors commonly associated with aging and aging associated diseases (Hegde et al., 2023) where signaling mechanisms that originate at MERCs may be an Achillis heel to these degenerative processes.

Under certain conditions and despite the above-described quality control mechanisms, the mitochondrial import machineries can be clogged by mitochondrial protein precursors. The mechanisms of protein clogging can be diverse and include the naturally occurring mutations discussed before, such as ANT1 alleles (Simoncini et al., 2017; Coyne et al., 2023) or mutations in the TOM70 receptor (Wei et al., 2020), and/or the excessive transcription-translation of mitochondrial precursors (Weidberg and Amon, 2018). Unclogging and clearance mechanisms are therefore needed, the components of which are analogous to those found in ERAD which are extensively reviewed elsewhere (Wiseman et al., 2022). In yeast, two mechanisms control the removal of non-imported precursors, the Mitochondrial Compromised Import Response (mitoCPR) (Weidberg and Amon, 2018) and mitochondrial protein translocation-associated degradation (mitoTAD) (Martensson et al., 2019) which differ in their mechanism of action. While the mitoCPR removes substrates on the surface of mitochondria (Weidberg and Amon, 2018), the mitoTAD removes arrested precursors from the TOM pore (Martensson et al., 2019). The mitoCPR is activated upon saturation of the import machineries and involves the receptor Tom70 which associates with Cis1 and the AAA-ATPase Msp1 which associate with the proteasome to promote the degradation of non-imported precursors (Weidberg and Amon, 2018). Instead, the mitoTAD requires the recruitment of Ubx2 that targets the clogged TOM complex and recruits the AAA-ATPase Cdc48 (Ye et al., 2001; Twomey et al., 2019) to subsequently unclog and degrade the toxic precursor (Martensson et al., 2019). p97/VCP (Valosin-containing protein) and ATAD1 (ATPase family AAA domain containing 1) are the mammalian orthologs of yeast Cdc48 and Msp1, respectively, and are also stress activated and participate in the removal of mistargeted proteins from mitochondria (Ye et al., 2001; Meyer et al., 2012; Chen et al., 2014). A limiting aspect in understanding protein clearance from mitochondria resides in the absence of some mammalian orthologs that need to be discovered explored.

## Lipid trafficking and membrane dynamics at the MERCs

**FIGURE 2:**
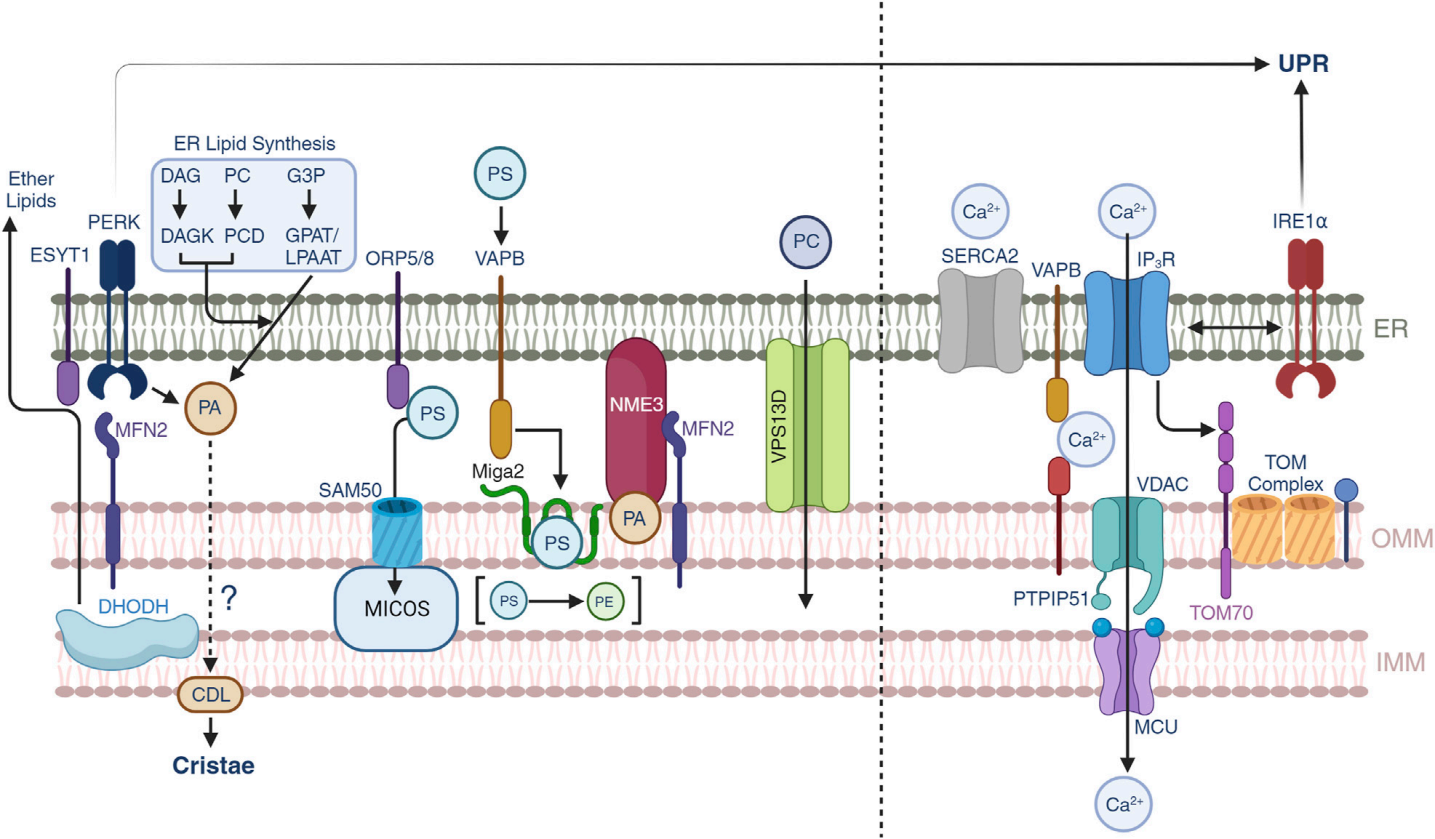
Lipid and calcium trafficking through the mitochondrial-ER contacts. Lipid trafficking is possible through subsets of protein-protein interactions between the ER and OMM. Phosphatidic acid (PA) is generated by three different pathways in the ER, yet under conditions of ER stress, PERK can also generate PA which is supplied to mitochondria to synthesize cardiolipin (CDL). Mitochondrial DHODH activity can also crosstalk to the ER to generate ether lipids that can modulate mitochondrial IMM compositions. Phosphatidylserine transport is allowed through multiple pathways including ORP5/8-SAM50-MICOS, MFN2, and VAPB-MIGA2. NME3 can be attracted by clusters of PA molecules and participate in mitochondrial membrane dynamics. VPS13D provides a structural hydrophobic tunnel which is thought to transport lipid species across the ER and mitochondria. Calcium trafficking allows mitochondrial function and the release from the ER to mitochondrial is controlled by SERCA2 and IP_3_R channels. Calcium can be transported through the VAPB-PTPIP51 and the IP_3_R-VDAC-MCU pathways. Non-canonical roles of IRE1α suggest a control over the latter. Recently, TOM70 has been found to also participate in the signaling of calcium to mitochondria.

The ER is a central component of the lipid synthesis machinery for the cell and supplies lipids and lipid precursors to other organelles in the cell including mitochondria (Balla et al., 2019). There is growing interest in understanding the metabolic pathways of lipid synthesis and the contribution of these metabolites to pathophysiological processes (Bennett et al., 2021; Mao et al., 2021). The protein and lipid environment shapes the biophysical nature of the membranes, their interactions with other organelles and the biochemical processes that segregate from the other membrane compartments (Balla et al., 2019). At the MERCs, the MAMs define a landscape of unique protein contacts that provide support for the transfer of lipids to mitochondria where the ISR is a fundamental part through non-canonical pathways. In this section we will provide insights into the regulation of lipid transport at the MERCs and its connection with membrane dynamics (Figure 2, left).

Both mitochondria and ER are dynamic organelles that undergo continuous cycles of membrane remodeling. Protein and lipids act in concert to shape mitochondria and ER and provide their functionality. The ER is constituted by a single lipid bilayer which is constituted by ∼50% phosphatidylcholine (PC), ∼30% phosphatidylethanolamine (PE), ∼15% phosphoinositol (PI) and ∼5% phosphatidylserine (PS) (van Meer et al., 2008). Interestingly, the ER is able to synthesize PC, PE, PI and PS, but also other non-ER or underrepresented membrane components in large amounts including phosphatidic acid. (PA), ceramides and galactoceramides, cholesterol and triglycerides. The discrepancy between the membrane composition at the ER and the lipids synthesized arouse the hypothesis of a lipid gradient that is transferred to other organelles including mitochondria (Balla et al., 2019). ER morphology fluctuates in response to environmental stressors where lipid stress can trigger conformational changes in the ER. This implies that nutrient or fatty acid stress can impact the ER stress responses and mitochondrial-ER communication (Arruda et al., 2014). Lipid-remodeling in the ER is dynamic and the resulting morphology fluctuates between two distinct domains; the ER sheets and tubules (Wang and Rapoport, 2019). Here, different classes of proteins work to stabilize the curvature of tubules, and facilitate the fusion of tubules into sheets, respectively (Wang and Rapoport, 2019). The tubules are stabilized by a class of membrane proteins termed reticulons (Rtns) and Receptor expression enhancing proteins (REEPs) that are sufficient and necessary to generate this morphology (Voeltz et al., 2006). Structural details of these protein-protein interactions remain to be elucidated, but closely-spaced transmembrane regions with amphipathic helixes have been proposed to introduce positive curvature of the ER membrane (Hu et al., 2008). Tubule membrane fusion promotes the formation of ER sheet networks that are facilitated by membrane-bound GTPases called alastins (ATLs, Sey1 in yeast) (Hu et al., 2009; Wang and Rapoport, 2019). Here, ATLs localized on distinct proximal tubules dimerize through interactions of their GTPase domains followed by conformational changes and fusion of neighboring lipid bilayers (Wang and Rapoport, 2019). These dynamic tubular-fusion events are important in supporting mitochondrial dynamics, and in fact, mutations in ATLs and REEPs have been associated with the onset of spastic paraplegia (SP), a neurodegenerative disorder characterized by axonal dysfunction (Hubner and Kurth, 2014). Interestingly, SP neurons also exhibit mitochondrial dysfunction with fragmented and bioenergetically depleted mitochondrial networks, consistent with the requirement of ER morphological dynamics for these processes (Hubner and Kurth, 2014).

Mitochondria are constituted by two distinct lipid bilayers, OMM and IMM, described in previous sections. The biochemical lipid composition differs between them (Funai et al., 2020). The OMM is composed by ∼50% PC, 30% PE, 10% PI and ∼10% PS, PA, cardiolipin (CL) and lysophospholipids. In contrast, the IMM consist of ∼40% PC, 35% PE, 15% CL, 5% PI and ∼5% PS, phosphoglycerol and lysophospholipids. These differences derive from the necessity of the IMM to create invaginations known as cristae that harbor the respiratory complexes and create sharp curvatures to compartmentalize the internal cristae space. Lipids such as PE, CL, PA and lysophospholipids increase the curvature of membranes (Funai et al., 2020; Bennett et al., 2021). Mitochondrial membranes undergo fusion and fission cycles that have been extensively reviewed (Giacomello et al., 2020; Quintana-Cabrera and Scorrano, 2023). Mitofusins 1 (MFN1) and 2 (MFN2) and the dynamin-like GTPase OPA1 cooperate to promote mitochondrial fusion while the dynamin related protein 1 (DRP1) promotes mitochondrial fission events. MFN1, DRP1 and OPA1 locate to the mitochondria while MFN2 can reside both ER and OMM membranes. MFNs provide a tethering platform that allows fission and fusion events (de Brito and Scorrano, 2008). In these processes the ER is an active regulator of mitochondrial membrane dynamics. For instance, during fission, the ER embraces the mitochondria and physically constricts them through the DRP1 protein in a process that consumes GTP (Adachi et al., 2020). Mitochondrial fusion is possible by the contacts stablished by MFNs that position two adjacent mitochondrial membranes tethered by the ER (Chen et al., 2003). Interestingly, physical interaction between MFN2 and PERK controls PERK activity (Munoz et al., 2013) and likely mitochondrial protein and lipid remodeling (Bobrovnikova-Marjon et al., 2012; Munoz et al., 2013; Perea et al., 2023). In the crossroad of protein-protein interactions, PE, PA and CL enhance mitochondrial fusion while diacylglycerols (DAG) and lysophosphatidic acid facilitate fission events (Frohman, 2015). Physiologically, mitochondrial elongation in fasted livers is an example of adaptation that likely facilitates fatty acid oxidation (Sohn et al., 2023).

Mitochondria are entirely reliant on the ER to provide the lipids or lipid precursors necessary to build up both OMM and IMM. PC cannot be synthesized in mitochondria and is imported from the ER, while PE cannot be directly imported from the ER and instead, needs to be generated in the mitochondria from ER-derived PS by the mitochondrial PS decarboxylase (PSD) (Balla et al., 2019). The synthesis of PS is possible through the PS synthases 1 and 2 that utilize PC and PE as substrates, respectively. Both, PC and PE species contribute to cellular adaptation in cells under nutrient stress. For instance, reductions in the activity of the mitochondrial dyhydroortate dehydrogenase (DHODH) signal to the peroxisome to induce ether lipid synthesis that is finalized in the ER (Bennett et al., 2021). These ether lipid species are able to stabilize respiratory supercomplexes and augment their activity increasing cellular fitness (Bennett et al., 2021). Therefore, the trafficking of PC and PS species from the ER to mitochondria ensures proper cellular adaptative responses. In yeast, the ERMES system facilitates the transport of PS and PC to mitochondria (Kawano et al., 2018). However, the absence of mammalian orthologs of the ERMES requires other mechanisms for lipid transport. VPS13D has been proposed as a bulk lipid transporter from the ER to mitochondria in yeast and mammals. VPS13D is single 500 kDa protein that tethers the ER and mitochondria (Guillen-Samander et al., 2021). Structurally, it forms a 160Å internal hydrophobic channel suitable for lipid transport (Li P. et al., 2020). Defects in VPS13D function result in severe ataxia (Dziurdzik et al., 2020).Oxysterol binding protein like 5 and 8 (ORP5/8) could participate in the delivery of PS to the Mitochondrial Contact Site and Cristae Organizing System (MICOS complex) (Monteiro-Cardoso et al., 2022). The MICOS complex is an active remodeler of cristae architecture which consists of the MIC60 subcomplex (MIC60, MIC19 and MIC25) and the MIC10 subcomplex (MIC10, MIC26 and MIC27) (Rampelt et al., 2017; Latorre-Muro et al., 2021). The subunits MIC26 and MIC27 have been associated with the transport of lipid species to mitochondria by unknown mechanisms (Anand et al., 2020). The regulation of MICOS complexes and cristae formation by PERK may coordinate the protein and lipid responses to facilitate cristae biogenesis (Bobrovnikova-Marjon et al., 2012; Latorre-Muro et al., 2021; Perea et al., 2023; Sassano et al., 2023). This would be facilitated by the interactions between PERK and ESYT1 that allow phospholipid transfer from the ER to mitochondria (Sassano et al., 2023). Structural studies have shown that Mitoguardin 2 (MIGA2) can transport phospholipids by interacting with the mitochondrial VAMP associated protein B (VAPB) with a strong preference for PS species (Kim et al., 2022). Therefore, multiple protein-protein contacts participate in the delivery and transport of PS species between the ER and mitochondria, albeit their mechanisms and/or targets remain still unsolved.

Another phospholipid which is crucial for mitochondrial architecture is phosphatidic acid (PA). PA is formed in the ER by three main pathways (Bobrovnikova-Marjon et al., 2012; Balla et al., 2019): phosphorylation of DAG by DAG kinases, hydrolyzation of PC by phospholipase D (PLD) or *de novo* synthesis from glycerol-3-phosphate and 2 fatty-acyl-CoA molecules by lysophosphatidic acid conversion by glycerol-3-phosphate acyltransferase (GPAT) which is acylated by lysophosphatidic acid acyltransferase (LPAAT). Importantly, the kinase domain of the ER-resident PERK which is exposed to the MAMs and OMM interface can act as a kinase for DAG transferring a phosphate group and forming PA under ER-stress conditions (Bobrovnikova-Marjon et al., 2012; Perea et al., 2023). Classically, the kinase domain of PERK phosphorylates eIF2α and controls protein translation by activating ATF4 (Harding et al., 1999; Harding et al., 2000). However, a non-canonical role of the PERK kinase domain provides a direct connection between the ISR and lipid synthesis. Aside from being an integral component of membranes, PA is a precursor for cardiolipin (CL) synthesis which exclusively occurs in mitochondria (Sustarsic et al., 2018). CL is highly abundant in the inner leaflet of IMM and greatly increases membrane curvature, as discussed above (Funai et al., 2020). CL is fundamental for OXPHOS activity and has deep physiological consequences on metabolic balance (Sustarsic et al., 2018). CL depletion upon ablation of cardiolipin synthase in brown adipocytes results in severe cold sensitive phenotype, obesity and insulin resistance (Sustarsic et al., 2018). PA transport to mitochondria is possible through VAPB which interacts with PtPiP51 and participate in the transport of PA (Yeo et al., 2021). The VAPB-PTPIP51 tethering system also participates in ER-mitochondrial calcium homeostasis (De Vos et al., 2012). It is unclear whether the PERK-MFN2 axis (Munoz et al., 2013) could participate in the transport of PA (Bobrovnikova-Marjon et al., 2012; Perea et al., 2023), although MFN2 can participate in the transport of other phospholipids such as PS (Hernandez-Alvarez et al., 2019). OMM PA can signal to cytoplasmic Nucleotide diphosphate kinase 3 (NDK3, or NME3 gene) via its N-terminal hydrophobic domain (Su et al., 2023). The recruitment is possible through the PA synthesizing enzyme PLD6 which favor the hexamerization in a process that may involve the presence of MFNs (Chen et al., 2019). In cells, nutrient deprivation increases the recruitment of NME3 and mitochondrial fusion in agreement with fasting-induced mitochondrial elongation in livers (Sohn et al., 2023). Therefore, lipids are signaling molecules that constitute a hydrophobic solvent that defines cellular components and separates chemical reactions and need to be synchronized with the protein machineries. The capacity to selectively recruit non-mitochondrial-ER proteins under specific cellular conditions illustrates how the surrounding protein environment define the function and dynamics of MERCs and cellular adaptive responses.

## Calcium signaling and mitochondrial-ER communication

In addition to cellular protein and lipid homeostasis, the ER is a vital organelle in the storage and distribution of calcium throughout the cell. Calcium flux between intracellular compartments generates a voltage-gradient across membranes that drives numerous signaling processes (Berridge et al., 2000). The ER accommodates a large range of Ca^2+^ concentrations at resting state between 100 and 800 µM. This compares to ∼100–200 nM in mitochondria, an organelle with particular high affinity for Ca^2+^, that can be stimulated to accommodate >20-fold resting state Ca^2+^ concentration to facilitate signaling events (Berridge et al., 2000). For example, Ca^2+^ coordinates cyclic AMP, nitric oxide (NO) synthase, phosphatidylinositol-3-OH kinase (PI(3)K), mitogen-activated protein kinases (MAPK), and is also involved in the feedback regulation of its own activity through modulating the function of different inositol-3-phosphates (Berridge et al., 2000). Calcium signaling is also tissue specific, where it plays differential roles in respiration, fertilization, embryogenesis, differentiation, proliferation, transcription factor activation, and apoptosis—processes that are extensively reviewed elsewhere (Berridge et al., 2000). Calcium flux at the MERCs plays an important role in responding to changes in intracellular calcium concentrations, which are regulated by ER-resident I_3_PR and SERCA, along with mitochondrial membrane voltage-dependent anion channels VDAC and mitochondrial calcium uniporters (MCU) (Berridge et al., 2000). Here, we will illustrate how Ca^2+^ at the MERCs is regulated by non-canonical roles of the components of the ISR and its roles in physiology and disease (Figure 2, right).

During brown fat thermogenesis, uncoupling protein 1 (UCP1) generates heat by uncoupling mitochondrial respiration and ATP synthesis (Bennett et al., 2022a). Interestingly, the inducible thermogenic beige fat utilizes a UCP1-independent mechanism of thermogenesis that employs the sarco/endoplasmic reticulum Ca^2+^-ATPase 2b (SERCA2b) and ryanodine receptor 2 (RyR2) (Ikeda et al., 2017). In the absence of UCP1, SERCA2b and RyR2 cooperate in the flux of calcium between the ER and mitochondria, a process that generates heat when uncoupled with ATP. Here, increased Ca^2+^ uptake into the mitochondria, along with enhanced glycolysis in beige adipocytes support the bioenergetic requirements of the cell through the TCA cycle and the electron transport chain (Ikeda et al., 2017). This Ca^2+^ futile cycle is relevant in diseases such as obesity and diabetes, where alterations in Ca^2+^ have been reported in the liver and pancreas (Park et al., 2010; Fu et al., 2011; Santulli et al., 2015). Specifically, diet-induced obesity is associated with defects in Ca^2+^ handling and reduced SERCA2 levels, providing further evidence of this futile cycling being an important metabolic adaptation to nutrient stress (Ikeda et al., 2017). The importance of MAMs in the pathophysiology of diabetes is further illustrated with the observation that MAMs are dysfunctional in diabetic livers, and its integrity is required for insulin signaling (Arruda et al., 2014; Tubbs et al., 2014). Taken together, Ca^2+^ futile cycles represent a mitochondria-ER communication that maintains cellular integrity under nutrient stress, and represent an actionable target in associated diseases. This non-canonical mechanism of cold-induced thrermogenesis at the MERCs can be reconciled with the PERK-OGT-TOM70 mediated protein import axis as noted previously (Latorre-Muro et al., 2021).

ER-mitochondrial calcium homeostasis impinges on non-canonical roles of the UPR sensors (Hetz et al., 2020). The UPR plays important roles in cellular homeostasis, and its dysfunction, and/or aberrant activation, is associated with pathology including metabolic diseases, type-two diabetes, obesity, aging, and many others (Hetz et al., 2020). Classically understood to be activated upon protein stress, recent evidence suggests that UPR sensors ATF6 and IRE1α play roles in sensing and responding to Ca^2+^ stress (Carreras-Sureda et al., 2019; Burkewitz et al., 2020). In *C. elegans*, ATF6 is hyperactivated upon aging which results in the overexpression of calreticulin and subsequent ER-calcium retention (Burkewitz et al., 2020). This pathological Ca^2+^ imbalance leads to impaired calcium signaling, reduced mitochondrial Ca^2+^ uptake via VDAC and MCU, and concomitant mitochondrial dysfunction (Burkewitz et al., 2020). In this regard, ATF6-KO is sufficient to reduce ER calreticulin levels to promote Ca^2+^ flux to the mitochondria and activate mitochondrial metabolism to delay the mitochondrial decline associated with aging (Burkewitz et al., 2020). Although this phenomenon is currently only observed in worms, the communication between the ER and mitochondria and its role in the aging process can be appreciated in the context of Ca^2+^ signaling under other stress conditions where UPR mis-regulation results in pathophysiology. Additionally, IRE1α senses and responds to calcium at MAMs by controlling mitochondrial Ca^2+^ uptake (Carreras-Sureda et al., 2019). Here, IRE1α acts as a scaffold where it interacts with IP_3_Rs dictating Ca^2+^ uptake into mitochondria at MAMs. Consistent with this, overexpression of IP_3_Rs in IRE1-KO cells restored calcium uptake into the mitochondria (Carreras-Sureda et al., 2019). Interestingly, this interaction is independent of IRE1α kinase or RNAase activity, positioning this protein-protein interaction unique to canonical UPR activity and XBP1 signaling. Importantly, mice deficient in IRE1α had altered mitochondrial metabolism along with defective glucose tolerance consistent with a diabetic phenotype (Carreras-Sureda et al., 2019).

Mis-regulation of Ca^2+^ flux and signaling is a common feature of oncogenesis (Chen and Cubillos-Ruiz, 2021). Rapid proliferation and cell division exhibited by tumors results in nutrient stress, with subsequent defects in protein glycosylation and folding in the ER. These resulting ER stress responses promote defects in Ca^2+^ handling by SERCA2 (Chen and Cubillos-Ruiz, 2021). In these contexts, nutrient and calcium stress are sensed by and impact mitochondrial function, as mentioned above, and hence likely play an important role in signaling and responding to these events. Specifically, cancers associated with obesity exhibit maladaptive accumulation of long-chain fatty acids in the ER (Chen and Cubillos-Ruiz, 2021). Here, alterations in ER membrane fluidity and structure result in defects in Ca^2+^ storage capacity and ER stress (Chen and Cubillos-Ruiz, 2021). In addition to nutrient stress, reactive oxygen species (ROS) generation, considered ubiquitous in cancers, result in ER lipid peroxidation that similarly affect Ca^2+^ homeostasis (Chen and Cubillos-Ruiz, 2021). In this context, mitochondria represent the predominant source of cellular ROS and their proximity to ER membranes may provide a direct target of ROS in cancers that lead to oncogenic Ca^2+^ dysfunction and ER-stress. These representative examples provide clear evidence of the importance of Ca^2+^ in cancer and how the ER and mitochondria respond and communicate to its dysfunction.

### Therapeutical targeting of mitochondrial-ER communication

Mitochondrial-ER communication is at the intersection of fundamental biological processes and associates with the development of several metabolic diseases described before including, obesity, T2D, cancer, or neurological disorders. Initial approaches targeting the ISR were focused on the treatment of pathological processes where protein accumulation and aggregation occur. The discovery of other roles for the three main branches of the ISR (ATF6, IRE1α and PERK) controlling multiple aspects of mitochondrial function and physiology have highlighted the importance of targeting mitochondrial-ER communication as emerging therapies.

Disruption of mitochondrial-ER functionality has been successfully applied in cancer. FDA approved proteasomal inhibitor Bortezomib, Nelfinavir, or Atazanavir increase the amounts of unfolded proteins and activate ER stress responses that result lethal to cancer cells (Nawrocki et al., 2005; Pyrko et al., 2007). Interestingly, PERK inhibition has been shown to alleviate the symptoms of Alzheimer’s disease (Ma et al., 2013; Rozpedek et al., 2019). Although the activation of PERK responses is physiologically beneficial for mitochondrial function (Latorre-Muro et al., 2021) and biogenesis (Kato et al., 2020), it may lead to pro-apoptotic responses that can damage neurons and exacerbate neurological disorders (Rozpedek et al., 2019). As such the beneficial or deleterious effects of UPR responses hinge on the degree of activation.

Tetracyclines, the FDA approved class of antibiotics that inhibit bacterial and mitochondrial protein synthesis, are pharmacologically beneficial for the treatment of mitochondrial diseases in cells and in mice (Perry et al., 2021; Ronayne et al., 2023). Intermediate doses of tetracyclines that partially inhibit mitochondrial translation result in a mitohormetic cell survival response that impinges on the mitoribosome quality control factors and the UPR as described above (Perry et al., 2021; Ronayne et al., 2023). This treatment paradigm illustrates a novel and durable strategy that corrects downstream inflammatory signaling that is exacerbated in mitochondrial mutants. Importantly, neuro-inflammation, the characteristic pathologic transition in preclinical mouse models of mitochondrial disease, is reversed with tetracyclines (Perry et al., 2021). Hence, repurposing of FDA approved tetracyclines for mitochondrial disease therapy may be promising.

In age-related processes such as obesity or T2D that occur with mitochondrial-ER miscommunication, strategies to activate mitochondrial-ER communication need to be clinically explored. Current evidence shows that mitochondrial-ER activation can have diverse outcomes depending on the cellular and physiological context. For instance, high calorie feeding increases the amounts of mitochondrial-ER contacts in the liver (Arruda et al., 2014). Likewise, PERK activation increases respiratory function in mouse livers (Balsa et al., 2019). Both responses can be understood as a physiological adaptation in response to nutrient overloads. However, synthetic induction of mitochondrial-ER contacts *in vivo* is not protective and exacerbates the obese phenotype (Arruda et al., 2014) highlighting the tight balance between the chemical and physical nature of mitochondrial-ER contacts and the resolution of stress responses. Therefore, the therapeutical applications of mitochondrial-ER activation require fine tuning and need further investigation in order to provide efficient therapies for metabolic disorders.

## Conclusion

The mechanisms that allow mitochondrial-ER communication provide an effective evolutionary adaptive cellular fitness. Herein, we have covered the regulatory mechanisms that regulate protein synthesis, trafficking and degradation focusing at the ER-OMM interface. Despite the current knowledge of some of these processes in mammals, their connection with pathological disorders or even adaptive processes is still elusive. Importantly, some of the essential regulatory mechanisms that have been studied in non-mammalian systems do not have orthologs, yet they participate in quality control processes such as protein insertion or unclogging that are fundamental. To address these questions, studying the influence of ISR component activation in shaping the ER membrane-OMM landscape will provide insights into how stress responses crosstalk to mitochondria to promote cell survival and/or protect against diseases. A common signature upon defective activation of the ISR branches is the association with the development of age-related diseases such as obesity, diabetes, cancer or neurogenerative processes. This agrees with the current models where mitochondrial-ER communication vanishes over time and impinges on the importance of studying how the reversal of these interferences can protect from the development of these pathologies.

Along with the protein-protein interactions, metabolic fluxes are central for the buildup of cellular structures such as mitochondria, which undergo profound cristae remodeling and biogenesis during stress responses such as fasting in liver or cold stimulation in brown adipocytes. In such cases, calcium and lipid trafficking results essential for the function and remodeling of mitochondria respectively. To date, it is highly unclear how lipid species are transported from the ER to mitochondria, whether the known pathways can reverse the flow of lipids and which forces drive such transport. In mammals, VPS13D has been postulated as major transporter of lipids due to the hydrophobicity of its central tunnel. However, many other protein contacts have been shown to participate in the delivery of phospholipids to mitochondria even though the species are not well known. Calcium flux between the mitochondria and ER has been established through the protein-protein interactions and activation of ER and mitochondrial Ca^2+^ channels. However, in cases where there are not direct mitochondrial ER contacts (ex. SERCA2), mechanisms by which ER-resident Ca^2+^ is directed specifically to the mitochondria to promote respiration requires further investigation. Additionally, the role of Ca^2+^ in the development of cancer has been appreciated, but pharmacological targeting has not yet been effectively explored in the context of clinical oncogenesis. Future work on the study of ER and mitochondrial Ca ^2+^ and lipid compositions across pathophysiological conditions in cells and *in vivo* is necessary to precisely understand lipidome remodeling in the context of cellular and tissue adaptation and the molecular basis of disease.
